# Characterizing causality in cancer

**DOI:** 10.7554/eLife.53755

**Published:** 2019-11-29

**Authors:** Elena Rondeau, Nicolas Larmonier, Thomas Pradeu, Andreas Bikfalvi

**Affiliations:** 1 University of BordeauxBordeauxFrance; 2 ImmunoConceptCNRS UMR 5164BordeauxFrance; 3 IHPSTCNRS UMR 8590ParisFrance; 4 LAMC-INSERM U1029BordeauxFrance

**Keywords:** philosophy of biology, causality, carcinogenesis, metastasis

## Abstract

Philosophers have explored the concept of causality for centuries. Here we argue that ideas about causality from philosophy can help scientists to better understand how cancerous tumors grow and spread in the body. After outlining six characteristics of causality that are relevant to cancer, we emphasize the importance of feedback loops and interactions between tumor-cell-intrinsic and tumor-cell-extrinsic factors for explaining the formation and dissemination of tumors.

## Introduction

'What causes cancer?' is perhaps the central question in cancer research. This question can be understood in various ways, including mechanistically (how does a tumor grow and spread within an organism?) and etiologically (what are the factors that initiate and favor cancer development?). In this article we focus on mechanistic questions, combining philosophical and biological insights to identify six key characteristics that can be used to explore causality in the context of cancer. We will not discuss etiological questions, though these remain of interest to philosophers working on biology and medicine (see, for example, Vineis et al., 2017).

Philosophy of science has a long tradition of investigating the different aspects of causality, including causal inference, probability, counterfactuals and manipulability (Woodward, 2003). Here, we build on this work – especially on cases in which philosophical analyses have been applied to cancer (Bertolaso, 2011; Plutynski, 2018) – to revisit the question 'what causes cancer?' in terms of the following six characteristics:

**Multicausality**: many different factors influence cancer initiation and dissemination.
**Causal variability**: the factors that influence the formation and dissemination of tumors can vary significantly according to tumor type, local context, level of analysis, and the unique history of each tumor.
**Causal necessity and/or sufficiency**: some factors may, by themselves, be sufficient to influence cancer initiation and dissemination, or they may be just one factor among many. (Indeed, it is now recognized that most cancers result from a combination of factors).
**Causal intricacy**: the factors that influence the formation and dissemination of tumors interact in complex ways, so much so that it is difficult to attribute a specific causal role to a given factor.
**Sequence-dependent causality**: cancer is an evolving process, with different factors having different roles at different stages.
**Spatially-situated causality**: factors may operate within the tumor microenvironment, or they may operate from a distance. Because we aim to identify where and when to intervene in order to prevent the disease (Woodward, 2003), clarifying the spatial location of causal factors is crucial (Laplane et al., 2018; Laplane et al., 2019).

In what follows we will first consider causality in the formation of tumors, and then go on to discuss causality in the dissemination of tumors. Two main points will stand out. First, despite a frequent focus on tumor-cell-intrinsic factors, in many cases it is the interaction between the intrinsic and the extrinsic factors that is important. Second, although it might seem natural to equate the causality of cancer with its temporal sequence (that is, with the different steps of cancer progression), it will in fact be necessary to distinguish causality from sequence because of the presence of feedback loops and because some causal connections might go 'backwards'.

## Causality in tumor formation

**Figure 1.**
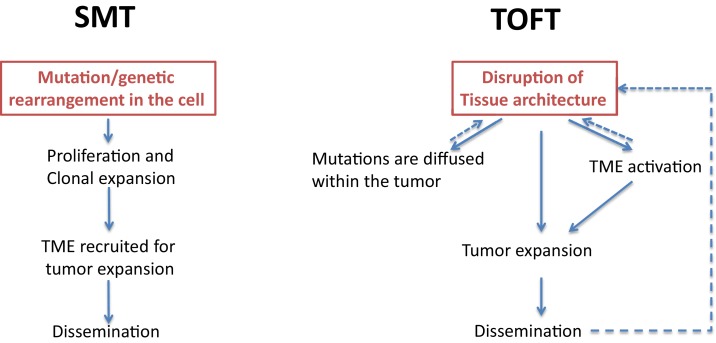
Two theories of tumor formation. In the somatic mutation theory (SMT; left) the default state of the cell is quiescence, and a genetic event in the cell triggers a unidirectional, irreversible and deterministic process that leads to tumor expansion and dissemination. In the tissue organization field theory (TOFT; right), the default state of the cell is proliferation, and a disruption of the tissue architecture leads to the diffusion of various mutations within the tumor and to the activation of the tumor microenvironment (TME). Through feedback mechanisms, this leads to further disruption of the tissue architecture, which promotes tumor expansion and dissemination.

There are two main explanatory schemes for tumor formation (Figure 1): a tumor-cell-centric view, starting with an event within the cell which initiates an avalanche of secondary events; and a tumor environment-centric view, which emphasizes the multiplicity of interactions in the tumor environment and the reversibility of many cancer-related events. Here we examine two theoretical frameworks for these two views.

In the somatic mutation theory, which centers on the tumor cells, the default state of a cell is quiescence, and a tumor forms as a result of genetic mutations in a single cell (Stratton et al., 2009). In this theory, causality is due to a single factor, and causal variability exists because different oncogenic drivers may be found in different tissues. A good example is the onset of hematological malignancies such as chronic myelogenous leukemia, with the production of an aberrant BCR-ABL fusion protein. In the somatic mutation theory, sequence-dependent causality and causal intricacy have minimal roles, and distant causality does not have any role. However, the introduction of sequences of mutations (such as those seen in multistage carcinogenesis) can increase the causal complexity associated with this theory.

The tissue organization field theory, on the other hand, centers on the tumor environment. This theory involves multiple causalities at various levels, as well as causal variability, causal intricacy, sequence-dependent causality and spatially-situated causality (Soto and Sonnenschein, 2011). The disruption of tissue architecture is the critical causal event in this theory, while mutations (which are randomly distributed), are a consequence of this disruption.

Although these two theories have their strengths and weaknesses, we believe that recent work tends to support the tissue organization field theory more than the somatic mutation theory, at least for some cancers. In pancreatic cancer, for example, it has been shown that tissue architecture plays a decisive role in modulating the phenotypes of tumor cells (Ligorio et al., 2019). Furthermore, cancer-associated driver mutations are distributed in most of the organs that are perfectly normal (Yizhak et al., 2019). Other examples include the way interactions between the tumor, its microenvironment and the immune system have a key role in cancer progression, and the way tumor vascularization is critical for awakening dormant tumors and for tumor expansion. Evidence is also emerging for causal interactions between the tumor and its broader 'organismal environment', such as the microbiota or the central nervous system (Laplane et al., 2019). However, the tissue organization field theory also makes a causal connection between the disruption of the tissue architecture and mutations in the tumor cells, a concept that is debatable insofar as mutations may also occur randomly.

## Causality in tumor dissemination

**Figure 2.**
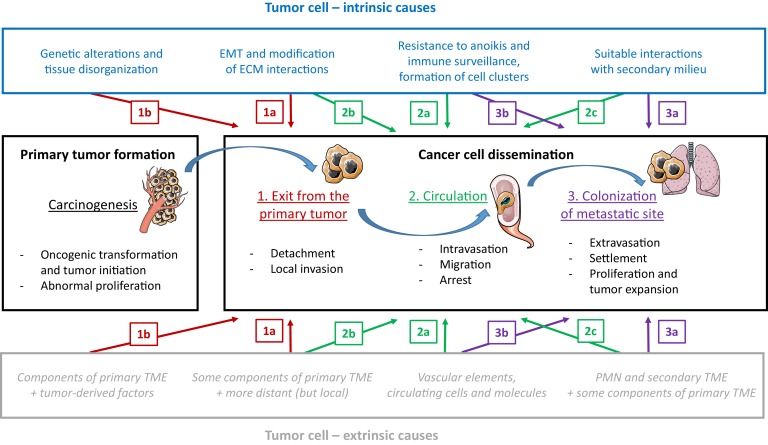
The influence of tumor-cell-intrinsic and tumor-cell-extrinsic factors on tumor formation and the dissemination of tumor cells. The main steps in the progression of cancer (carcinogenesis and the three steps of dissemination) are shown in the center panels, along with the key tumor-cell-intrinsic factors (top; blue text) and tumor-cell-extrinsic factors (bottom; grey text) that influence progression. We will use step 2 of dissemination to explain the different types of causal relationships proposed in the figure. An example of an intrinsic factor acting at a given step (a type 'a' event) is the formation of clusters of tumor cells to enhance migration efficiency (2a, top), and an example of an extrinsic factor is the protection provided by vascular elements against immune attack and physical stress, during the same step (2a, bottom). Events during a given step can also exert an influence on a later step (type 'b' events): for instance, the initiation of the epithelial-to-mesenchymal transition (EMT) during step 1 of dissemination triggers the possibility of long-distance circulation in step 2 (2b, top). Events during a given step can also exert an influence on an earlier step (type 'c' events): for instance, the elements in the secondary tumor microenvironment (TME), which are part of step 3 of dissemination, also act as attractors for cancer cells during step 2 (2c, bottom). PMN: pre-metastatic niche.

Tumor dissemination is a multistep process that involves the passage of tumor cells from the primary site to metastatic sites located in one or more distant organs. It is characterized by the following events (Figure 2): i) exit from the primary tumor, ii) circulation through the bloodstream or lymphatic system, and iii) colonization of the metastatic site (if the cells settle there). A comprehensive understanding of all these steps – which involve interactions between various tumor-cell-intrinsic and tumor-cell-extrinsic factors – is crucial to the design of more efficient therapies.

Historically, Paget's 'seed and soil' metaphor has been used to describe how dissemination results from favorable interactions between circulating tumor cells (the seed) and the specific microenvironments they encounter (the soil). This analogy is still used today, although we know much more about what happens at the cellular and molecular levels (Langley and Fidler, 2011). Here we discuss causality during the three stages of dissemination, and show how some or all of the six characteristics mentioned above are involved.

### Exit from the primary tumor

Different causal factors, both intrinsic and extrinsic to the tumor cell, are involved in the two processes that make up this step – the detachment of cancer cells from the tumor, and their journey towards an accessible blood or lymph vessel (which, depending on the size and vascularization of the primary tumor, may require invasion of the surrounding tissue, also known as local or loco-regional invasion). In particular, the epithelial-to-mesenchymal transition (EMT) enables cancer cells to lose intercellular adherence and acquire the mesenchymal properties that foster local invasion and migration. Other tumor-cell-intrinsic mechanisms are involved, such as aberrant intracellular signaling (e.g., EGFR amplification or truncation), the loss of adhesion molecules (such as E-cadherin), the expression of transcription factors that regulate the transition, and the production of proteins that degrade the extracellular matrix.

Tumor-cell-extrinsic factors are also crucial for local invasion (Quail and Joyce, 2013). For instance, stromal cells produce pro-migratory factors that are required for tumor cell motility and for remodeling the extracellular matrix, as well as a signaling molecule called TGF-β (transforming growth factor beta) that stimulates the EMT. Conversely, interactions between stromal cells and cancer cells undergoing the EMT may influence the phenotypic and functional features of immune cells (Chockley and Keshamouni, 2016). This illustrates causal intricacy, and more specifically here, a reciprocity between two types of causal actors in invasion.

Local invasion also displays characteristics of causal necessity and/or sufficiency and sequence-dependent causality. For instance, the effects of TGF-β on cancer progression are highly dose-dependent and they may vary from step to step: for example, TGF-β can act as a tumor suppressor in one step, and then help to stimulate the EMT in a later step (Bachman and Park, 2005). Moreover, some epithelial cells can enter the circulation without undergoing an EMT: this is possible due to the phenomena of clustered migration (where transitioned and non-transitioned cells move together) and the remodeling of the primary microenvironment by mesenchymal cells. This means that the EMT may be considered permissive rather than necessary for metastasis (Jolly et al., 2017).

### Circulation

To enter the bloodstream or the lymphatic system – a process known as intravasation – a cancer cell must cross an endothelial barrier. Again, this involves both tumor-cell-intrinsic factors (such as the expression of adhesion molecules and permeability factors) and tumor-cell-extrinsic factors (such as interactions with myeloid cells and endothelial cells, or feedback loops involving small signaling molecules called cytokines; Su et al., 2014). An instance of causal intricacy here is the fact that the tumor vasculature can undergo its own EMT (called an endoEMT) and favor the transmigration of tumor cells into the circulation by disrupting the endothelial cell barrier during intravasation.

Once in the bloodstream, the survival of circulating tumor cells, their arrest in the capillaries, and their extravasation into the metastatic tissues, depend on the intrinsic properties of the cells (such as resistance to anoïkis and their ability to avoid immune surveillance) and on various aspects of their local environment (Strilic and Offermanns, 2017). The cells can, for example, protect themselves by forming clusters, which may be homotypic (i.e., exclusively composed of tumor cells) or heterotypic (i.e., they can also contain neutrophils, myeloid suppressor cells or platelets; Szczerba et al., 2019).

When metastatic cells reach the vessels at the secondary site, their exit from the circulation relies on both intrinsic changes (such as the reversal of the EMT for cells that have transitioned) and extrinsic factors (such as another EndoEMT, this time linked to tumor cell extravasation). Neutrophil extracellular traps also have an important role in removing tumor cells so that they can undergo extravasation (Cools-Lartigue et al., 2013), as does the nature of the surface molecules expressed by endothelial cells at the metastatic sites.

### Colonization of the metastatic site

The successful colonization of a secondary site depends on the early establishment of a 'pre-metastatic niche' (i.e., a local environment that is favorable to the seeding of circulating cancer cells). Again, both tumor-cell-intrinsic factors (such as cytokines and exosomes derived from the primary tumor; Tung et al., 2019) and tumor-cell-extrinsic factors (e.g., subsets of cells derived from the bone marrow; Gao et al., 2019) are involved. Moreover, the types of causal explanations for site-specific seeding are multiple and diverse. Some organs (such as the growth factor–enriched bone microenvironment) may manifest a certain predisposition for welcoming tumor cells. Other metastases rely mainly on specific organ chemokines binding to cancer cell receptors (such as CXCL12 binding to CXCR4; see also Liotta, 2001). Recent research also suggests that organ-specific angiocrine signaling from endothelial cells may be an important site-specific mechanism for metastasis.

In addition, the facilitating role of pro-colonization factors may be attributed to both tumor-cell-extrinsic factors (such as immune cells of the pre-metastatic niche) and the cancer cells themselves. Indeed, metastatic cancer cells may cooperate with early migrating cells, whose 'inefficient' seeding could nevertheless create conditions that are more favorable for later waves of cells (Bidard et al., 2008).

Organ colonization by cancer cells can lead to two different fates: tumor expansion, or a period of dormancy followed by reactivation. Tumor expansion at a secondary site requires many interactions that are similar to those required for primary tumor growth, though these interactions are adapted to the new environment. Moreover, cross-talk between different metastatic sites may influence the development of secondary tumors.

Cancer cells and tumor-cell-extrinsic factors can also travel between the different environments involved in metastasis. It is likely that cancer cells leave the primary tissue early in tumor development, and that they may later migrate from established metastases in the case of further seeding at new sites (Gundem et al., 2015). Metastatic cells and molecules can also travel back to the primary site, where they may contribute to continued growth of the primary tumor, the growth of new blood vessels, and the recruitment of stromal cells to the tumor (Kim et al., 2009). This phenomenon is called 'self-seeding' and, like the feedback loops discussed previously, is another example of how the causality involved in cancer is more complex than suggested by the classic sequential view of tumor formation and dissemination.

## Conclusion

Exploring the multi-dimensional nature of causality in cancer – especially dimensions that tend to be neglected, such as sequence-dependent causality and spatiality-dependent causality – has the potential to improve our understanding of how the disease originates, develops and spreads. In particular, we draw attention to seven points:

- The explanatory power of conceptual frameworks: two philosophies of cancer, the tumor-cell-centric view and the environment-centric view, have critically shaped our physio-pathological understanding of cancer (Bissell and Radisky, 2001). Current research could benefit from a thorough consideration of the intricacy of these two frameworks and their complementarity.
- The distinction between deterministic and stochastic causal explanations: while deterministic explanations may be sufficient in some cases, stochastic explanations will be required in others.
- The relative strengths of the various causes or types of causes involved in cancer initiation and dissemination: if hierarchies of causal influences could be established, they could be used to prioritize targets for drug discovery research.
- The existence of different causal explanations at different stages of the disease: identifying these different explanations, and determining if they are connected or not, will benefit researchers.
- The organ-specific nature of metastatic colonization is usually an example of causal intricacy: in some cases, it will be possible to identify a single causal explanation, but most cases will require multiple explanations, because they result from a variety of interacting causes.
- The nature of dormancy: how do dormant states differ from the normal physiological state, and how does causality intervene?
- Causalities can be nonlinear: a complete understanding of cancer is likely to involve various feedback and feed-forward loops.

A greater awareness of the complexity of causality will, we strongly believe, lead to a deeper understanding of the disease by philosophers, scientists and clinicians alike.

## Note

This Feature Article is part of the Philosophy of Biology collection.

## References

[bib1] Bachman KE, Park BH (2005). Duel nature of TGF-β signaling: tumor suppressor vs. tumor promoter. Current Opinion in Oncology.

[bib2] Bertolaso M (2011). Hierarchies and causal relationships in interpretative models of the neoplastic process. History and Philosophy of the Life Sciences.

[bib3] Bidard FC, Pierga JY, Vincent-Salomon A, Poupon MF (2008). A "class action" against the microenvironment: do cancer cells cooperate in metastasis?. Cancer and Metastasis Reviews.

[bib4] Bissell MJ, Radisky D (2001). Putting tumours in context. Nature Reviews Cancer.

[bib5] Chockley PJ, Keshamouni VG (2016). Immunological consequences of epithelial-mesenchymal transition in tumor progression. Journal of Immunology.

[bib6] Cools-Lartigue J, Spicer J, McDonald B, Gowing S, Chow S, Giannias B, Bourdeau F, Kubes P, Ferri L (2013). Neutrophil extracellular traps sequester circulating tumor cells and promote metastasis. Journal of Clinical Investigation.

[bib7] Gao Y, Bado I, Wang H, Zhang W, Rosen JM, Zhang XH (2019). Metastasis organotropism: redefining the congenial soil. Developmental Cell.

[bib8] Gundem G, Van Loo P, Kremeyer B, Alexandrov LB, Tubio JMC, Papaemmanuil E, Brewer DS, Kallio HML, Högnäs G, Annala M, Kivinummi K, Goody V, Latimer C, O'Meara S, Dawson KJ, Isaacs W, Emmert-Buck MR, Nykter M, Foster C, Kote-Jarai Z, Easton D, Whitaker HC, Neal DE, Cooper CS, Eeles RA, Visakorpi T, Campbell PJ, McDermott U, Wedge DC, Bova GS, ICGC Prostate Group (2015). The evolutionary history of lethal metastatic prostate cancer. Nature.

[bib9] Jolly MK, Ware KE, Gilja S, Somarelli JA, Levine H (2017). EMT and MET: necessary or permissive for metastasis?. Molecular Oncology.

[bib10] Kim MY, Oskarsson T, Acharyya S, Nguyen DX, Zhang XH, Norton L, Massagué J (2009). Tumor self-seeding by circulating cancer cells. Cell.

[bib11] Langley RR, Fidler IJ (2011). The seed and soil hypothesis revisited--the role of tumor-stroma interactions in metastasis to different organs. International Journal of Cancer.

[bib12] Laplane L, Duluc D, Larmonier N, Pradeu T, Bikfalvi A (2018). The multiple layers of the tumor environment. Trends in Cancer.

[bib13] Laplane L, Duluc D, Bikfalvi A, Larmonier N, Pradeu T (2019). Beyond the tumour microenvironment. International Journal of Cancer.

[bib14] Ligorio M, Sil S, Malagon-Lopez J, Nieman LT, Misale S, Di Pilato M, Ebright RY, Karabacak MN, Kulkarni AS, Liu A, Vincent Jordan N, Franses JW, Philipp J, Kreuzer J, Desai N, Arora KS, Rajurkar M, Horwitz E, Neyaz A, Tai E, Magnus NKC, Vo KD, Yashaswini CN, Marangoni F, Boukhali M, Fatherree JP, Damon LJ, Xega K, Desai R, Choz M, Bersani F, Langenbucher A, Thapar V, Morris R, Wellner UF, Schilling O, Lawrence MS, Liss AS, Rivera MN, Deshpande V, Benes CH, Maheswaran S, Haber DA, Fernandez-Del-Castillo C, Ferrone CR, Haas W, Aryee MJ, Ting DT (2019). Stromal microenvironment shapes the intratumoral architecture of pancreatic cancer. Cell.

[bib15] Liotta LA (2001). An attractive force in metastasis. Nature.

[bib16] Plutynski A (2018). Explaining Cancer: Finding Order in Disorder.

[bib17] Quail DF, Joyce JA (2013). Microenvironmental regulation of tumor progression and metastasis. Nature Medicine.

[bib18] Soto AM, Sonnenschein C (2011). The tissue organization field theory of cancer: A testable replacement for the somatic mutation theory. BioEssays.

[bib19] Stratton MR, Campbell PJ, Futreal PA (2009). The cancer genome. Nature.

[bib20] Strilic B, Offermanns S (2017). Intravascular survival and extravasation of tumor cells. Cancer Cell.

[bib21] Su S, Liu Q, Chen J, Chen J, Chen F, He C, Huang D, Wu W, Lin L, Huang W, Zhang J, Cui X, Zheng F, Li H, Yao H, Su F, Song E (2014). A positive feedback loop between mesenchymal-like cancer cells and macrophages is essential to breast cancer metastasis. Cancer Cell.

[bib22] Szczerba BM, Castro-Giner F, Vetter M, Krol I, Gkountela S, Landin J, Scheidmann MC, Donato C, Scherrer R, Singer J, Beisel C, Kurzeder C, Heinzelmann-Schwarz V, Rochlitz C, Weber WP, Beerenwinkel N, Aceto N (2019). Neutrophils escort circulating tumour cells to enable cell cycle progression. Nature.

[bib23] Tung KH, Ernstoff MS, Allen C, Shu S (2019). A review of exosomes and their role in the tumor microenvironment and host-tumor "macroenvironment". Journal of Immunological Sciences.

[bib24] Vineis P, Illari P, Russo F (2017). Causality in cancer research: a journey through models in molecular epidemiology and their philosophical interpretation. Emerging Themes in Epidemiology.

[bib25] Woodward JF (2003). Making Things Happen: A Theory of Causal Explanation.

[bib26] Yizhak K, Aguet F, Kim J, Hess JM, Kübler K, Grimsby J, Frazer R, Zhang H, Haradhvala NJ, Rosebrock D, Livitz D, Li X, Arich-Landkof E, Shoresh N, Stewart C, Segrè AV, Branton PA, Polak P, Ardlie KG, Getz G (2019). RNA sequence analysis reveals macroscopic somatic clonal expansion across normal tissues. Science.

